# Supplementary material for: Towards Thermally Reversible Networks Based on Furan-Functionalization of Jatropha Oil
Source: Molecules. 2020 Aug 10;25(16):3641. doi: 10.3390/molecules25163641 (PMC7465891; doi:10.3390/molecules25163641)
Supplement: Supplementary file 1 [file molecules-25-03641-s001.pdf]

Supplementary Information : Furan-functionalization of jatropha oil

Frita Yuliati, Peter J. Deuss, Hero J. Heeres, and Francesco Picchioni

1. Proton NMR spectra demonstrating the removal of excess furfurylamine.

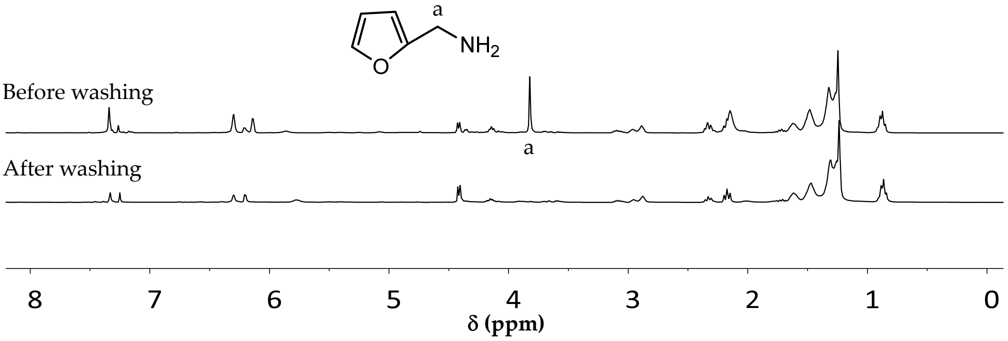

Figure S1. NMR spectra of reaction product before and after washing

2. Statistical analysis of reactions with LiBr

The experiments were performed according to Table 1, with measured responses described in Table S1. This set of data was analyzed by using the analysis of variance (ANOVA) method, using the Minitab 17 software package. The estimated effects and the coefficient of the equation for data models are given in Table S2, Table S4, and Table S6. Analysis of variance for each of the responses are given in Table S3, Table S5, and Table S7. The residual plots of the responses are given in Figure S2, Figure S3, and Figure S4, while the pareto graphs of the standardized effects for the responses are given in Figure S5.

Table S1. Experimental design with temperature, molar ratio between furfurylamine and epoxides, reaction time, and LiBr loading as the factors / independent variables (coded units); ester and epoxide conversions and furans attached as the responses / dependent variables.

Std. Order	Temperature	Molar ratio	Time	LiBr loading	Ester conversion	Epoxide conversion	Furans attached
1	-1	-1	-1	-1	25%	22%	3%
2	1	-1	-1	-1	46%	52%	14%
3	-1	1	-1	-1	60%	15%	19%
4	1	1	-1	-1	65%	33%	22%
5	-1	-1	1	-1	33%	24%	8%
6	1	-1	1	-1	46%	75%	29%
7	-1	1	1	-1	61%	14%	20%
8	1	1	1	-1	76%	85%	51%
9	-1	-1	-1	1	23%	14%	4%
10	1	-1	-1	1	40%	41%	13%
11	-1	1	-1	1	60%	18%	19%
12	1	1	-1	1	79%	77%	33%
13	-1	-1	1	1	35%	33%	8%

Table S1 continued.

Std. Order	Temperature	Molar ratio	Time	Catalyst loading	Ester conversion	Epoxide conversion	Furans attached
14	1	-1	1	1	31%	98%	20%
15	-1	1	1	1	69%	19%	24%
16	1	1	1	1	64%	96%	56%
17	-1	-1	-1	-1	26%	20%	5%
18	1	-1	-1	-1	48%	54%	12%
19	-1	1	-1	-1	63%	18%	19%
20	1	1	-1	-1	70%	39%	24%
21	-1	-1	1	-1	36%	22%	9%
22	1	-1	1	-1	38%	69%	29%
23	-1	1	1	-1	61%	11%	21%
24	1	1	1	-1	79%	84%	53%
25	-1	-1	-1	1	20%	13%	3%
26	1	-1	-1	1	44%	53%	12%
27	-1	1	-1	1	59%	8%	22%
28	1	1	-1	1	76%	57%	34%
29	-1	-1	1	1	36%	35%	8%
30	1	-1	1	1	39%	100%	18%
31	-1	1	1	1	66%	16%	23%
32	1	1	1	1	76%	95%	58%

Table S2. Estimated Effects and Coefficients for Ester conversion (coded units)

**Term**	**Effect**	**Coef**	**SE Coef**	**T**	**P**
Constant		0.51523	0.008655	59.53	0
Temperature	0.11484	0.05742	0.008655	6.63	0
Molar ratio	0.32422	0.16211	0.008655	18.73	0
Time	0.02734	0.01367	0.008655	1.58	0.129
LiBr loading	-0.01016	-0.00508	0.008655	-0.59	0.564
Temperature*Molar ratio	-0.00703	-0.00352	0.008655	-0.41	0.689
Temperature*Time	-0.05078	-0.02539	0.008655	-2.93	0.008
Temperature*LiBr loading	-0.01328	-0.00664	0.008655	-0.77	0.451
Molar ratio*Time	-0.00078	-0.00039	0.008655	-0.05	0.964
Molar ratio*LiBr loading	0.02734	0.01367	0.008655	1.58	0.129
Time*LiBr loading	-0.00703	-0.00352	0.008655	-0.41	0.689

S = 0.0489591 PRESS = 0.116882
R-Sq = 95.12% R-Sq(pred) = 88.68% R-Sq(adj) = 92.80%

Table S3. Analysis of Variance for Ester conversion (coded units)

Source	DF	Seq SS	Adj SS	Adj MS	F	P
Main Effects	4	0.95326	0.953262	0.238315	99.42	0
Temperature	1	0.10551	0.105513	0.105513	44.02	0
Molar ratio	1	0.84094	0.840942	0.840942	350.83	0
Time	1	0.00598	0.005981	0.005981	2.5	0.129
LiBr loading	1	0.00083	0.000825	0.000825	0.34	0.564
2-Way Interactions	6	0.02882	0.028818	0.004803	2	0.111
Temperature*Molar ratio	1	0.0004	0.000396	0.000396	0.17	0.689
Temperature*Time	1	0.02063	0.02063	0.02063	8.61	0.008
Temperature*LiBr	1	0.00141	0.001411	0.001411	0.59	0.451
Molar ratio*Time	1	0	0.000005	0.000005	0	0.964
Molar ratio*LiBr	1	0.00598	0.005981	0.005981	2.5	0.129
Time*LiBr loading	1	0.0004	0.000396	0.000396	0.17	0.689
Residual Error	21	0.05034	0.050337	0.002397		
Lack of Fit	5	0.03135	0.031353	0.006271	5.28	0.005
Pure Error	16	0.01898	0.018984	0.001187		
Total	31	1.03242				

Table S4. Estimated Effects and Coefficients for Epoxide conversion (coded units)

Term	Effect	Coef	SE Coef	T	P
Constant		0.44034	0.01382	31.85	0
Temperature	0.5028	0.2514	0.01382	18.18	0
Molar ratio	-0.02443	-0.01222	0.01382	-0.88	0.387
Time	0.21179	0.1059	0.01382	7.66	0
LiBr loading	0.08645	0.04322	0.01382	3.13	0.005
Temperature*Molar ratio	0.05551	0.02775	0.01382	2.01	0.058
Temperature*Time	0.15587	0.07793	0.01382	5.64	0
Temperature*LiBr loading	0.07228	0.03614	0.01382	2.61	0.016
Molar ratio*Time	-0.01863	-0.00931	0.01382	-0.67	0.508
Molar ratio*LiBr loading	0.0247	0.01235	0.01382	0.89	0.382
Time*LiBr loading	0.05061	0.02531	0.01382	1.83	0.081

S = 0.0782058   PRESS = 0.298235
R-Sq = 95.51%   R-Sq(pred) = 89.58%   R-Sq(adj) = 93.38%

Table S5. Analysis of Variance for Epoxide conversion (coded units)

Source	DF	Seq SS	Adj SS	Adj MS	F	P
Main Effects	4	2.44586	2.44586	0.61147	99.98	0
Temperature	1	2.02244	2.02244	2.02244	330.67	0
Molar ratio	1	0.00478	0.00478	0.00478	0.78	0.387
Time	1	0.35886	0.35886	0.35886	58.67	0
LiBr loading	1	0.05979	0.05979	0.05979	9.78	0.005
2-Way Interactions	6	0.28894	0.28894	0.04816	7.87	0
Temperature*Molar ratio	1	0.02465	0.02465	0.02465	4.03	0.058
Temperature*Time	1	0.19435	0.19435	0.19435	31.78	0
Temperature*LiBr loading	1	0.04179	0.04179	0.04179	6.83	0.016
Molar ratio*Time	1	0.00278	0.00278	0.00278	0.45	0.508
Molar ratio*LiBr loading	1	0.00488	0.00488	0.00488	0.8	0.382
Time*LiBr loading	1	0.02049	0.02049	0.02049	3.35	0.081
Residual Error	21	0.12844	0.12844	0.00612		
Lack of Fit	5	0.08966	0.08966	0.01793	7.4	0.001
Pure Error	16	0.03877	0.03877	0.00242		
Total	31	2.86324				

Table S6. Estimated Effects and Coefficients for Furans attached (coded units)

Term	Effect	Coef	SE Coef	T	P
Constant		0.216455	0.0061	35.49	0
Temperature	0.163884	0.081942	0.0061	13.43	0
Molar ratio	0.187813	0.093906	0.0061	15.39	0
Time	0.110817	0.055409	0.0061	9.08	0
LiBr loading	0.01036	0.00518	0.0061	0.85	0.405
Temperature*Molar ratio	0.04079	0.020395	0.0061	3.34	0.003
Temperature*Time	0.077302	0.038651	0.0061	6.34	0
Temperature*LiBr loading	0.002045	0.001022	0.0061	0.17	0.868
Molar ratio*Time	0.031297	0.015648	0.0061	2.57	0.018
Molar ratio*LiBr loading	0.038683	0.019341	0.0061	3.17	0.005
Time*LiBr loading	-0.01719	-0.0086	0.0061	-1.41	0.173

S = 0.0345062 PRESS = 0.0580599
R-Sq = 96.45% R-Sq(pred) = 91.76% R-Sq(adj) = 94.76%

Table S7. Analysis of Variance for Furans attached (coded units)

Source	DF	Seq SS	Adj SS	Adj MS	F	P
Main Effects	4	0.596154	0.596154	0.149038	125.17	0
Temperature	1	0.214863	0.214863	0.214863	180.45	0
Molar ratio	1	0.282188	0.282188	0.282188	237	0
Time	1	0.098244	0.098244	0.098244	82.51	0
Catalyst loading	1	0.000859	0.000859	0.000859	0.72	0.405
2-Way Interactions	6	0.08332	0.08332	0.013887	11.66	0
Temperature*Molar ratio	1	0.013311	0.013311	0.013311	11.18	0.003
Temperature*Time	1	0.047805	0.047805	0.047805	40.15	0
Temperature*Catalyst loading	1	0.000033	0.000033	0.000033	0.03	0.868
Molar ratio*Time	1	0.007836	0.007836	0.007836	6.58	0.018
Molar ratio*Catalyst	1	0.011971	0.011971	0.011971	10.05	0.005
Time*Catalyst loading	1	0.002364	0.002364	0.002364	1.99	0.173
Residual Error	21	0.025004	0.025004	0.001191		
Lack of Fit	5	0.023211	0.023211	0.004642	41.43	0
Pure Error	16	0.001793	0.001793	0.000112		
Total	31	0.704478				

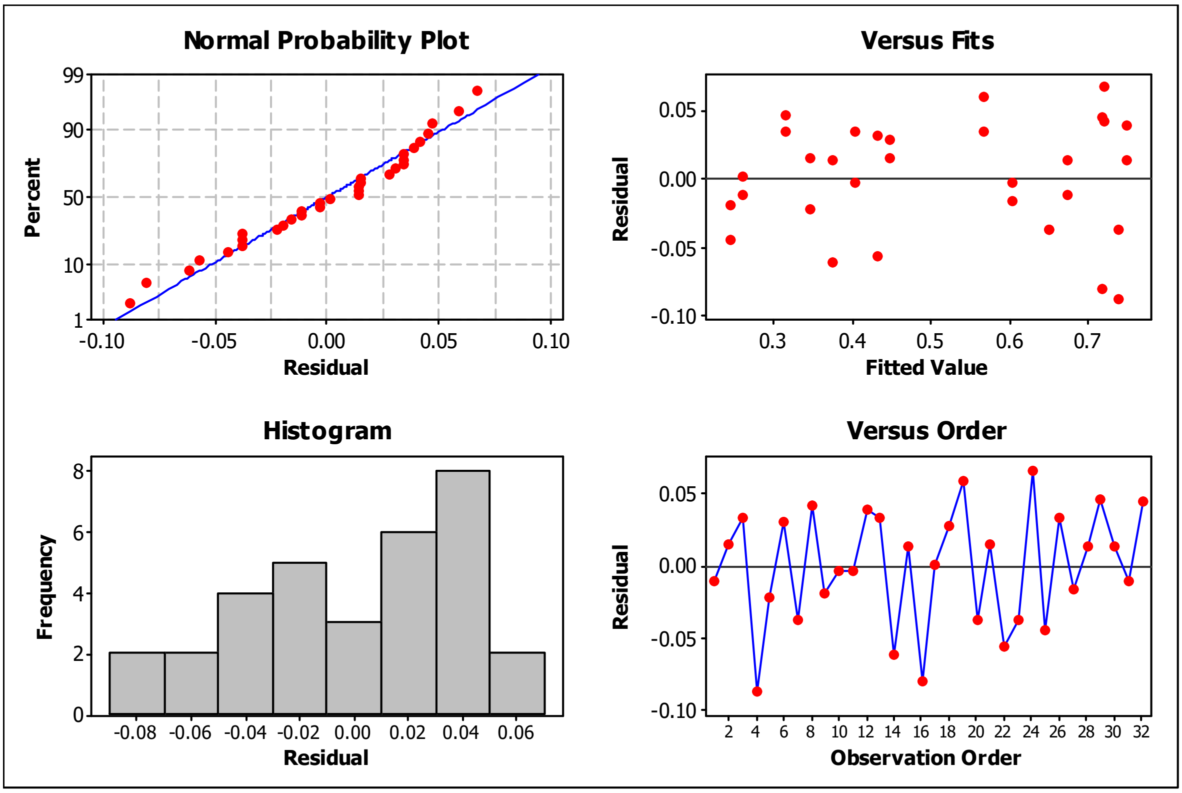

Figure S2. Residual plots for ester conversion

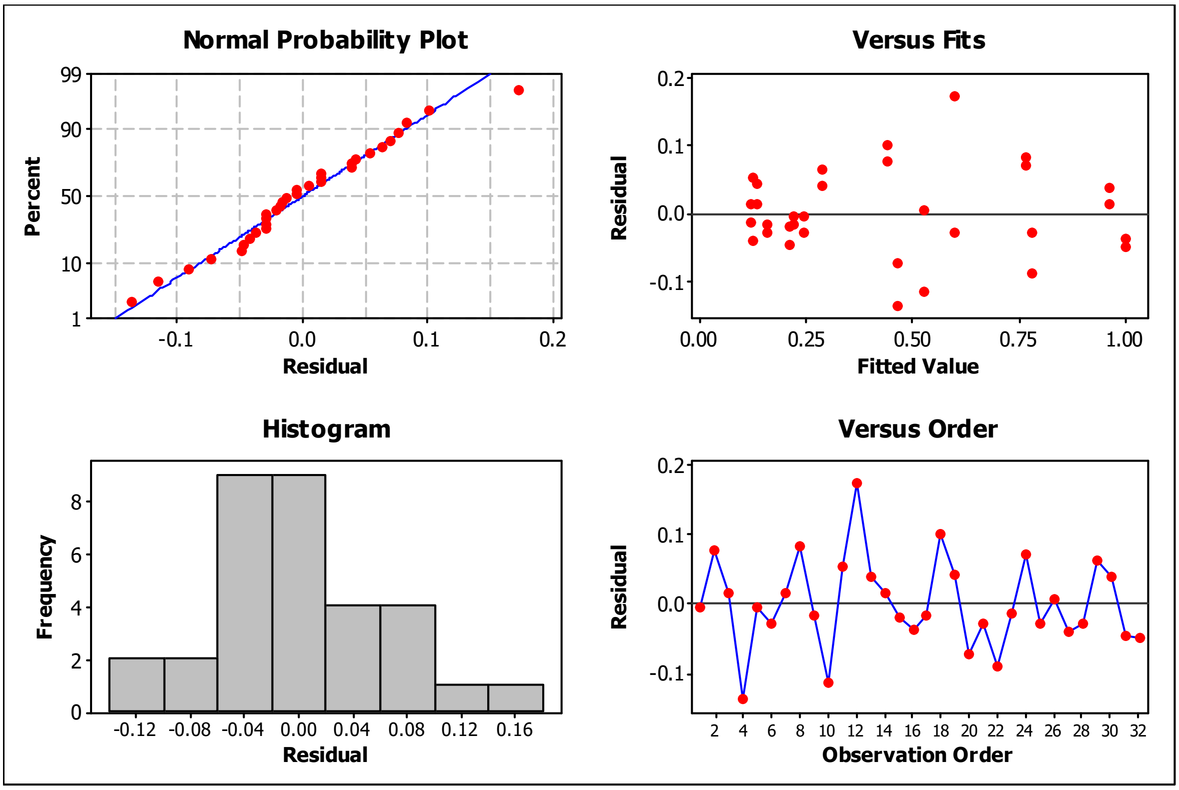

Figure S3. Residual plots for epoxide conversion

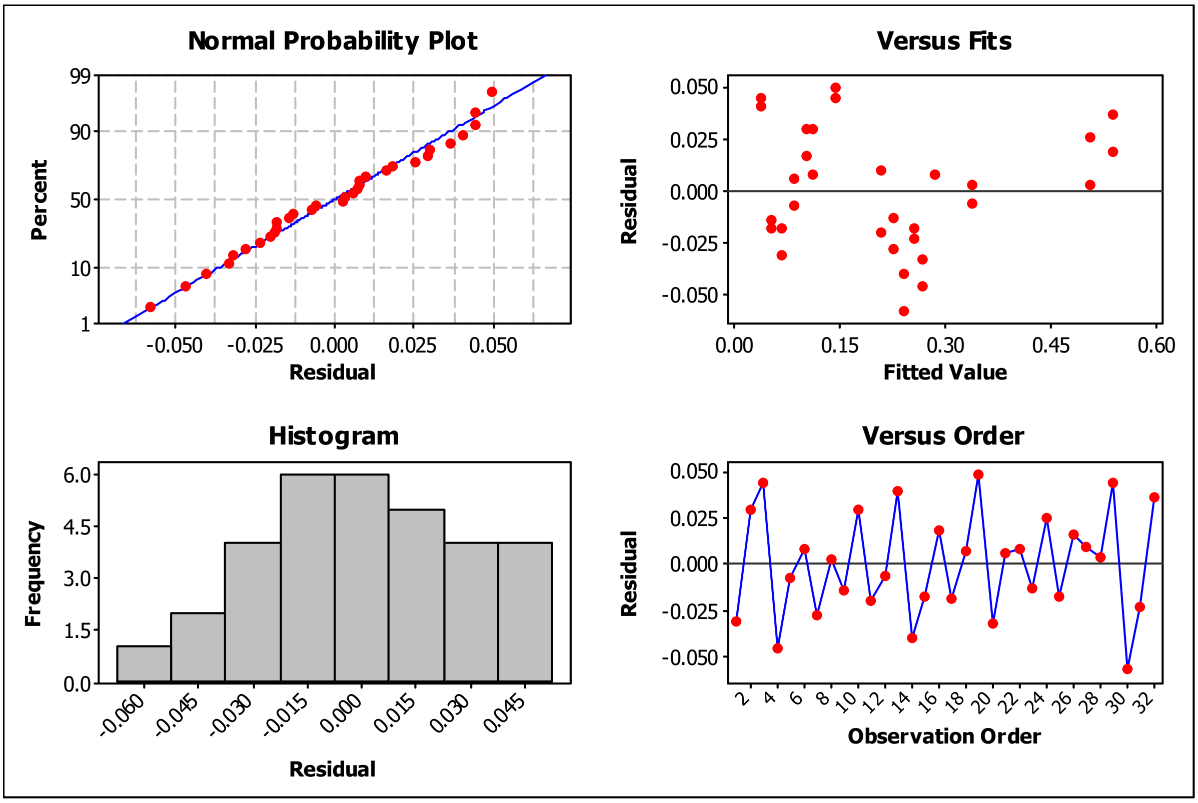

Figure S4. Residual plots for furans attached

(a) Pareto chart of standardized effect, response is ester conversion

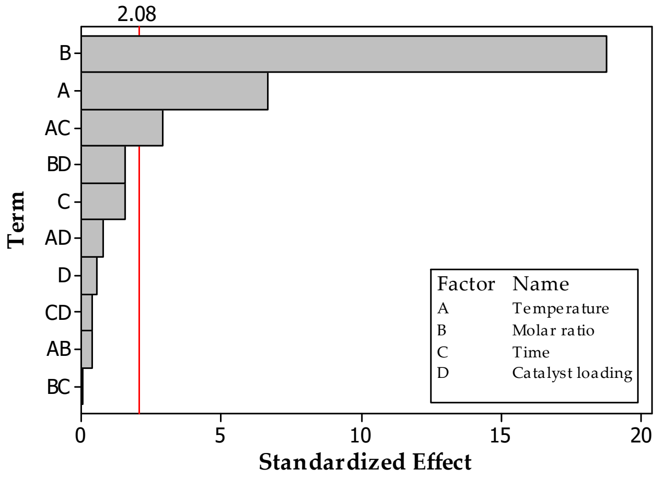

(b) Pareto chart of standardized effect, response is epoxide conversion

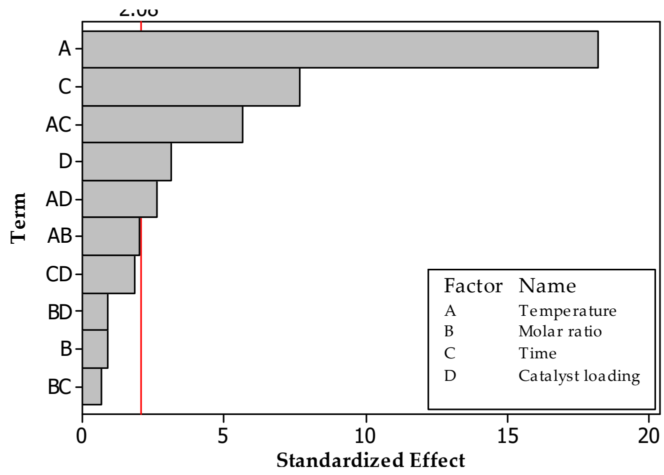

(c) Pareto chart of standardized effect, response is furans attached

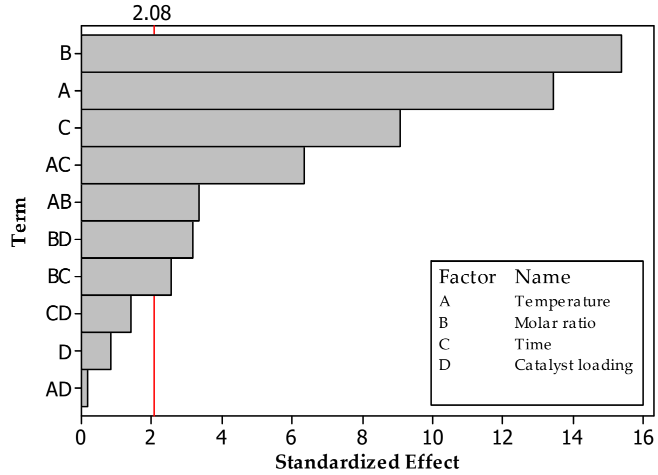

Figure S5. Pareto charts of standardized effects of each responses

3. Side reactions

Side reaction was a prevalent phenomenon observed in this study. The number of furans attached to the modified triglycerides was always lower than the number of esters and epoxides converted. Water could be present as traces from the epoxidation of the oil. We suspected hydrolysis as the side reaction and conducted some experiments to test the hypothesis. Epoxidized oil was mixed with water (2 wt. %) and LiBr (50 mol %) and stirred at 80 ^{o}C for 24 h. The product was analyzed by using ^{1}H NMR.

NMR spectra of the products show that new signals appear in the mixture containing LiBr (Figure S3). The broad signals located at δ 1.85-2.20 ppm appeared strongly in this mixture (spectrum ii) and can be attributed to the hydroxyl groups produced when esters are converted or when epoxides are hydrolyzed. The areas under the triglyceride ester signals (δ 2.30 ppm) and epoxide signals (δ 2.82 – 3.18 ppm) were smaller than those in the epoxidized oil, indicating that esters and epoxides were hydrolyzed. The broad signals at δ 1.85-2.20 ppm were found in many experiments in this study, especially in those conducted at 80 ^{o}C. This data revealed that hydrolysis was responsible for the relatively low number of furans attached.

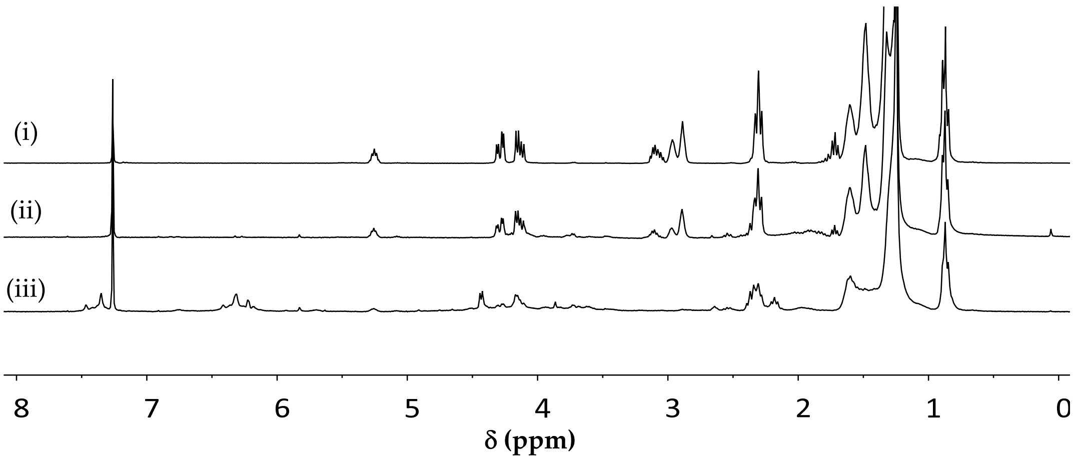

Figure S6. ^{1}H NMR spectra indicating the occurrence of hydrolysis: (i) epoxidized jatropha oil; (ii) mixture of epoxidized oil, water, and LiBr heated at 80 ^{o}C for 24 hours; (iii) the experiment {100,24,1,100}.

4. Measured number of incorporated furan

Table S8. The number of incorporated furan per molecule, as a percentage of the total number of esters and epoxides originally existed in the epoxidized oil

Temperature (^{o}C)	Molar ratio	Time (h)	Furan : (Original Ester + Epoxide), without catalyst	Furan : (Original Ester + Epoxide), With 50% LiBr
30	1:1	5	1%	4%
30	1:5	5	1%	19%
30	1:1	24	1%	8%
30	1:5	24	1%	20%
80	1:1	5	2%	12%
80	1:5	5	2%	32%
80	1:1	24	3%	19%
80	1:5	24	4%	54%
